# Bayesian methods for estimating injury rates in sport injury epidemiology

**DOI:** 10.1186/s40621-025-00583-z

**Published:** 2025-06-06

**Authors:** Avinash Chandran, Ben Lambert

**Affiliations:** 1 Datalys Center for Sports Injury Research and Prevention, Indianapolis, 46220 IN USA; 2 Department of Statistics, Oxford University, 24-29 St Giles, Oxford, OX1 3LB UK

## Abstract

**Background:**

The injury rate is a common measure of injury occurrence in epidemiological surveillance and is used to express the incidence of injuries as a function of both the population at risk as well as at-risk exposure time. Traditional approaches to surveillance-based injury rates use a frequentist perspective; here, we discuss the Bayesian perspective and present a practical framework on how to apply a Bayesian analysis to estimate injury rates. We estimated finescale injury rates across a broad range of categories for men’s and women’s soccer, applying a Bayesian methodology and using injury surveillance data captured within the National Collegiate Athletic Association Injury Surveillance Program from 2014/15–2018/19.

**Results:**

Through an iterative process of assessing model fidelity, we found that a negative binomial model was an effective choice for modeling surveillance-based injury rates. We also found differences between schools to be a key driver of variation in injury rates.

**Conclusions:**

Our findings indicate that the Bayesian framework naturally characterizes injury rates by modeling injury counts as outcomes of an underlying data-generation process that explicitly incorporates inherent uncertainty, complementing traditional frequentist approaches. Key benefits of the Bayesian approach in this context are the ability to test model suitability in a variety of methods, and to be able to generate plausible estimates with sparse data.

**Supplementary Information:**

The online version contains supplementary material available at 10.1186/s40621-025-00583-z.

## Background

Measures of occurrence are common across epidemiology and include the incidence rate, point prevalence, and prevalence proportion [1, 2]. The incidence rate is a powerful measure of outcome occurrence in epidemiology, as it expresses the occurrence of events as a function of the population at risk as well as at-risk exposure time [1]. Considering both the population and exposure time is important in this calculation as outcome events are inherently contingent upon not only the population under observation, but also the period of observation (when taken together, a composite measure of population and exposure time is often referred to as time spent in population) [1, 3]. In injury epidemiology, the injury rate is a naturally employed analog to the epidemiological incidence rate and has been widely used in various contexts [4–7]. Within sport injury epidemiology especially, injury rates computed on the basis of injury surveillance data have been widely used to quantify the incidence of specific injuries (e.g., sport-related concussion) in various athlete populations, or to assess overall injury patterns across various sports [8–12]. Such efforts have been vital in appraising the burden of injury in various athlete populations and are useful in informing playing rule changes and policy initiatives.

As in other variations of the incidence rate, the injury rate in sport injury epidemiology is computed as a measure of new injuries standardized by some measure of time spent in population. In US-based sport injury surveillance in particular, time spent in population is commonly expressed as athlete exposures– defined as one athlete participating in one exposure event (i.e., a practice session or competition) [4]. The injury rate is then computed as follows:

1 $$Rate= \frac{Total\: injuries}{Total\: Athlete\: exposures}$$

The injury rate is often standardized per 1,000 or 10,000 AEs [5, 8, 9, 13, 14]. Using this framework, it is also common practice to examine how and whether the injury rate varies systematically across risk groups using standard methods such as injury rate ratios [9, 12]. Importantly, traditional approaches to calculating surveillance-based injury rates use a frequentist perspective, relying exclusively on an observed sample of data collected in a given context (such as competition setting), or sport to produce estimates. However, this approach has limitations; for example, if no data were available for a given year within a multi-year study period, it would not be possible to estimate injury rates for the missing year under the frequentist framework. Alternatively, it is also possible to conceive of the injury rate as the outcome of a data generating process, which can be modeled– the approach taken in Bayesian inference.

We now consider a Bayesian perspective on estimating the injury rate. For conceptual purposes, we denote the injury rate by a parameter, $\theta$. The Bayesian inferential framework can then be summarized by Bayes’ rule as follows [15, 16]:

2 $$p\left(\theta|data\right)= \frac{p\left(data|\theta \right) \times p(\theta )}{p(data)}$$

The goal of Bayesian inference is characterized by $p\left(\theta|data\right)$ here, which represents the posterior probability distribution of the injury rate and summarizes the uncertainty in this quantity [16]. The first term in the numerator of the right side of Eq. (2) is referred to as the likelihood; for a fixed $\theta$, it represents the probability distribution over possible datasets. The likelihood is common to both the frequentist and Bayesian approaches; a fundamental distinction between the approaches is that the frequentist approach relies exclusively on this component.

The next term on the right side of Eq. 2, $p(\theta )$, represents the prior probability distribution and reflects the investigators’ beliefs characterizing the uncertainty across different values of $\theta$. This represents a choice made by investigators and is an added requirement in Bayesian inference, in contrast to the frequentist approach, which only requires specification of the likelihood. Priors can serve multiple purposes, draw on different sources of knowledge about a parameter, and their selection can meaningfully influence the resulting estimates. In subsequent sections, we describe our decision-making process for selecting priors in the context of a specific applied example. With that said, to illustrate how the choice of priors can affect posterior distributions, we may consider the following example of estimating the rate of lateral ligament complex tears (i.e., ankle sprains) in a National Collegiate Athletic Association (NCAA) sport such as women’s volleyball. [12] Recent publications indicate that ankle sprains are frequently reported injuries in NCAA sports, and the rate of ankle sprains in women’s volleyball is ~ 8 per 10,000 AEs. [12,17]

We suppose that there was a count of 84 injuries across 10,000 athlete exposures. We construct an example to model the count of injuries using a Poisson likelihood (with conjugate Gamma priors), and in Supplemental Fig. 1 we show the influence of the choice of prior on the posterior distribution of the injury rate. In the top panel of Supplemental Fig. 1, we show four prior probability densities; in the bottom panel, we show the impact that these priors have on the posterior distributions. Despite substantial differences in the shape and informativeness of the priors, the posterior distributions are similar– indicating that in this example, the data are sufficiently informative to dominate the influence of the prior. This illustrates that reasonable prior choices may have limited influence when the data are dense, although careful consideration of prior specification is important in Bayesian analysis as this may not hold in other settings where the data are less informative. While prior specification is a key consideration in Bayesian analysis, the use of priors provides important advantages. Indeed, incorporating prior beliefs enables the estimation of models with sparse data or complexities that frequentist methods alone cannot handle.

The denominator term in Eq. (2), $p(data)$, is the normalizing factor and represents the probability density of obtaining the dataset under analysis assuming the model and priors chosen by the analyst. In practical senses, this denominator in Bayesian inference is used to normalize the numerator in Eq. (2), so that the posterior distribution represents a valid probability distribution. It is also the source of some of the computational difficulty inherent in doing exact Bayesian inference. This is why approximate sampling methods, including Markov Chain Monte Carlo (MCMC), are often used to fit models in practice.

The Bayesian approach offers a varying perspective on the injury rate in sport injury epidemiology, providing a complementary parallel to the traditional frequentist approach. The purpose of this work is to leverage data collected within a multi-site, longitudinal sport injury surveillance system to present a Bayesian conceptualization of the injury rate and to offer practical considerations for applying this approach more generally. We present a systematic workflow and discuss various decision points in our analytical process, including our choice of models, prior distributions, and critical assessments of our inferences [18].

## Approach and findings

### Study data

We analyzed data captured within the National Collegiate Athletic Association (NCAA) Injury Surveillance Program (ISP) during the 2014/15–2018/19 academic years. The NCAA ISP is managed by the Datalys Center for Sports Injury Research and Prevention, an independent nonprofit research organization. The methods of the NCAA ISP have been reviewed and approved as an exempt study by the NCAA Research Review Board (RRB) and have been described previously [4]. Briefly, the system relies on a convenience sampling scheme with a rolling recruitment model for data collection. During the timeframe corresponding to the present work, Athletic Trainers (ATs) at participating institutions contributed exposure and injury data to the surveillance system using their institutional clinical Electronic Medical Record (EMR) systems. A common data elements export standard was used to extract relevant data from EMR records and patient identifiers were stripped as part of the submission process. Exported data were cycled through automated verification processes ensuring the consistency and fidelity of submitted data. ATs at participating institutions were able to report data for the sports of their choosing, and the number of reporting programs varied by sport and year.

Within the broader surveillance study, a reportable injury was defined as one that occurred due to participation in an organized intercollegiate practice or competition, and required medical attention by a team Certified Athletic Trainer or physician, regardless of time loss (i.e., regardless of whether/not the injury resulted in missed days of sport participation). For each injury, the ISP captured details regarding the injury (body part, diagnosis, etc.), and the circumstances surrounding the injury (injury mechanism, activity at time of injury, etc.). An exposure was defined as any team-sanctioned athletic activity in which student-athletes were participating and “exposed” to the risk of injury. The ISP captured exposure details (such as event type, surface type, etc.) and the number of athletes participating in each exposure event. The exposure data collected were used to estimate at-risk exposure time as athlete exposures (AEs)– defined as one athlete participating in one practice or competition event. For the present work, we chose to analyze injuries and exposures corresponding to men’s and women’s soccer reported within the 2014/15–2018/19 seasons. This represented 1,358 competition injuries (captured across 80,453 AEs) and 1,463 practice injuries (251,225 AEs) in men’s soccer, and 1,885 competition injuries (captured across 112,245 AEs) and 2,047 practice injuries (captured across 342,085 AEs) in women’s soccer.

## Model building and assessment

### Data visualizations and mathematical formulation

Based on the existing literature, we chose to analyze associations between injury rates and competition level (Division I, Division II, Division III), event type (practices, competitions), and injury diagnosis (categorized as sprains, strains, contusions, concussions, other) as primary explanatory variables of interest. As a primary step, we generated plots of injury rates across combinations of sport, event type, and injury diagnosis (Supplemental Fig. 2). Consistent with expectations based on existing injury surveillance literature, visual inspections revealed varying injury rate distributions by diagnosis and event type in both men’s and women’s soccer. For example, our data align with the published literature, indicating that the incidence rates of sprains and strains surpass those of concussions.[8–10, 12]. It has been previously reported, and our data confirm, that the rates of injuries by event type, and of specific injuries such as concussions, vary between men’s and women’s soccer athletes [8–10]. Accordingly, a decision was made to analyze men’s and women’s soccer separately within our modeling framework. To focus on the most common injury types, we restricted analyses to strains, sprains, contusions, and concussions.

To account for our count data, we first adopted a Poisson formulation of the following form to represent the count of injuries:

3 $${X}_{i} \sim Poisson({\lambda }_{i} \times {AEs}_{i})$$

where ${X}_{i}$ represents the observed count of injuries and ${\lambda }_{i}$ represents the injury rates per AE, for observation $i$.

### Model parametrization and specifying prior distributions

We modeled the relationship between injury rate and a range of explanatory factors:

4 $$\begin{aligned} {\lambda }_{i}&=\text{exp}(\gamma + {\alpha }_{Event[i]}+ {\beta }_{Division[i]}\\&\quad+ {\epsilon }_{Diagnosis[i]}+ {\kappa }_{Year[i]}) \end{aligned}$$

The parameters modeled in Eq. (4) (i.e., $\gamma, \alpha, \beta, \epsilon,\kappa$) indicate the strength of association between each explanatory variable and the injury rate, and we aim to estimate these associations. Our explanatory variables Event, Division, Diagnosis, etc. are categorical, and we allow one value of each parameter for each possible group within the category. For instance, $Event\left[i\right]$ could be one of either competition or practice. Bayesian analysis aims to estimate the posterior distribution over the parameters of interest based on the data and prior probability distribution. As described above (within the Background section), the choice of prior distributions is a demand additional to the choice of likelihood– the latter of which is the only requirement of frequentist inference.

Depending on the goals of the particular analysis, priors can serve many purposes. In some analyses, incorporating outside information is necessary in order to actually fit a model to data– in this sense, Bayesian inference is more powerful than frequentist inference because it allows inference to proceed with less data. Complex models of data generating processes, especially those incorporating physiological mechanisms, may involve many parameters, of which only a subset is known to high precision; some may be estimated from fitting the model to data; and some may not be inferable given the data. For this last group of parameters, prior distributions can be especially useful to constrain their ranges to plausible values, whilst still allowing the model to fit the data. This outside information may come from various sources such as prior studies, or expert knowledge. As a simple example– prior distributions for injury rates can be shaped to reflect the fact that rates must always be positive values and fall within plausible ranges (such as constraining the rate of catastrophic injury to lie between 0 and 0.01 per 1,000 athlete-exposures).

In analyses where either there is an emphasis on “letting the data speak for themselves”; or so-called objectivity, priors may be chosen that aim to minimize the effect of outside influences. For example, in a large-sample setting involving a well-studied outcome and strong, balanced data across groups, the observed data may be expected to dominate the posterior distribution, and minimally informative priors may be used. Another case would be if an imperative was placed on not influencing estimates through the use of information generated within a given study; for example, when assessing the efficacy of a novel vaccine. In this case, priors which were broad and unrestrictive could be preferable. There are many schools of thought on how such priors can be specified; a currently popular one is the concept of weakly informative priors [15]– these priors effectively aim to have minimal impacts on the bulk of the shape of the posterior and its central measures, for example, on the posterior median estimate. Their effects are then mainly on the tails of the posterior distribution, and weakly informative priors typically aim to truncate these distributions, which has benefits for the computational methods used in Bayesian inference (see *Fitting Model to Data*).

In our analysis, we use weakly informative priors to stabilize estimates in strata with limited data, and to reduce the risk of implausible parameter values– while preserving the influence of our observed data. The parameters in Eq. (4) all can be interpreted as relative rate multipliers when exponentiated: if ${\alpha }_{Event[i]}=0$, then that particular event type is not associated with a change in the injury rate relative to the base case and ${\text{exp}(\alpha }_{Event[i]})=1$. We specify wide Cauchy priors on these parameters: $\sim Cauchy(0, 10)$, for $\gamma, \alpha, \beta, \epsilon,\kappa$, which effectively represent a huge range of possible relative rate multipliers.

### Fitting the model to data

A key computational difference between frequentist and Bayesian inference is the computational methods used to fit models to data. In frequentist inference, typically a maximum likelihood estimate of the parameters is sought, which is obtained by using a computational method which maximizes the likelihood. In Bayesian inference, where the posterior distribution is sought, the predominant method used is computational sampling, and usually a specific variant of sampling known as MCMC is used.

We fit our models using Stan [19], a probabilistic programming language known for its adaptability and efficiency in handling complex statistical models. Taking as input the provided data, priors, and model structure, Stan generates samples from the posterior distribution of the parameter values. Like the results from throwing a dice many times can be used to approximate the probability distribution of the dice, samples from the posterior distribution can approximate it.

Like a roll of dice can only be approximated by its sequence of throws if it has been thrown enough times, a posterior distribution also needs a sufficient number of samples in order for it to be well-approximated. However, unlike repeated rolls from a fair die, initial samples obtained from MCMC do not yet come from the target posterior distribution—they are drawn from a different distribution, often influenced by initial values and the structure of the Markov chain. It is only after some time that the sequence of samples may settle down to a stable distribution, at which point the samples more closely approximate the posterior. This means that it is crucial to take care to diagnose whether or not the samples drawn have converged, and the standard approach to check this is to run multiple Markov chains [20]. By running multiple Markov chains, we maximize the chances of detecting a lack of convergence, and Stan runs 4 chains by default. We used diagnostic measures provided by Stan: $\widehat{R}$ and effective sample size (ESS), to evaluate model convergence (i.e., whether or not the Markov chain has reached its limiting distribution to approximate the posterior). $\widehat{R}$, or the Gelman-Rubin diagnostic, reflects if multiple sequences or “chains” of samples, generated independently, have reached a stable distribution, which is used to diagnose convergence to the posterior distribution [20]. If $\widehat{R} \le 1.01$ for all parameters, this is used as a threshold for MCMC convergence. ESS is a diagnostic measure that estimates the number of independent samples from the posterior distribution equivalent to the correlated samples obtained from MCMC methods. Generally, an ESS of at least 400 for all parameters is required to diagnose convergence [21]. Both of these diagnostics are essential for ensuring the dependability of inferences from a Bayesian analysis.

### Posterior predictive checks

In our analyses, posterior distributions of our parameters of interest (i.e., coefficients corresponding to each explanatory variable– characterizing their relationship with injury rates) can be used to compute predicted injury rates. By comparing these posterior predictive injury rates with the equivalents in the actual data, we can assess the goodness of the model fit to the data. These so-called posterior predictive checks are an essential part of the Bayesian workflow [18]. Bayesian analysis offers the flexibility to customize posterior predictive checks according to the specific analysis and model in question. Accordingly, in the present analysis, we considered the following posterior predictive checks across various contrasts of sport, event type, division, and diagnosis:

- Extreme values: Counting the fraction of simulated counts which exceed the maximum values in observed data.
- Proportions of zero cases: Counting the fraction of zero case counts and comparing to real zero counts.
- Plots of Actual vs. Model predicted injury rates: A comparison of how closely the predicted rates aligned with observed rates.
- Probability integral transforms: A comparison of the full distribution of the empirical data with that of the simulated data.

**Fig. 1:**
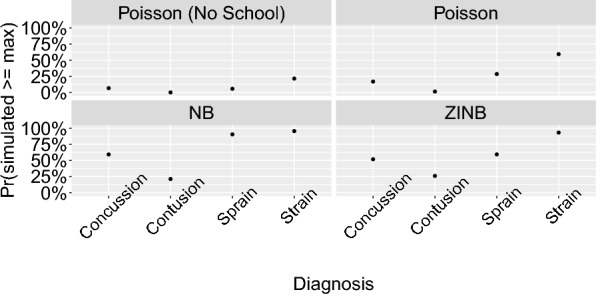
Maxima posterior predictive check. The example shown is for all diagnoses reported during competition events in Division I women’s soccer. Each point represents a specific diagnosis, with the y-axis indicating the proportion of simulations where the injury value exceeded the observed maximum, scaled as a percentage. The figure is faceted by model type to illustrate results from various model configurations. NB indicates the negative binomial model, and ZINB indicates the zero-inflated negative binomial model

The insights obtained from our posterior predictive checks played a crucial role in guiding our model selection process. Although the Poisson model demonstrated satisfactory performance in certain aspects of model fit, it fell short in others. The Poisson model inadequately handled extreme values, often generating probabilities near 0 that the simulated counts exceeded observed maximums across various diagnoses (Fig. 1). This result suggested that the Poisson model allowed for insufficient variation in the data generating process. This led us to reconsider the underlying factors that might be influencing the injury rates in our dataset. It has been previously reported that sports medicine resources and staffing may vary notably between institutions, and particularly across competition levels in the NCAA [22]. We therefore hypothesized that school reporting behaviors might play an important role, and we incorporated a school-level ”random effects” type term in the model:

5 $$\begin{aligned}{\lambda }_{i}&=\text{exp}(\gamma + {\alpha }_{Event\left[i\right]}+ {\beta }_{Division\left[i\right]}\\&\quad+ {\epsilon }_{Diagnosis\left[i\right]}+ {\kappa }_{Year\left[i\right]} + {\zeta }_{School[i]}) \end{aligned}$$

**Fig. 2:**
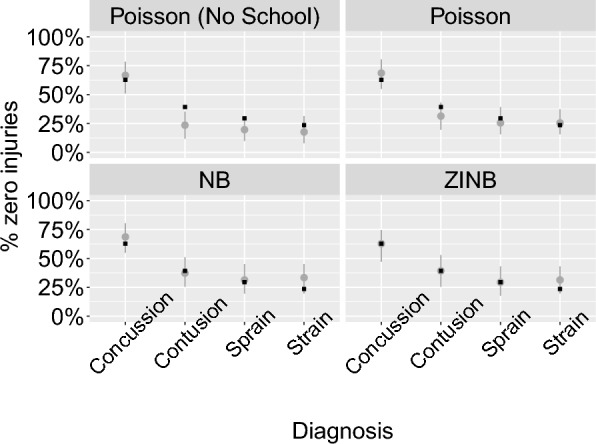
Observed proportion of zero injury values and the corresponding 95% uncertainty intervals for the simulated data. The example depicted shows all diagnoses reported during competition events in Division I men’s soccer. Each point represents a specific diagnosis, with the y-axis indicating the proportion of zero injuries, scaled as a percentage. The solid black circles denote observed proportions, while the grey lines indicate the 95% credible intervals from the simulated data. The figure is faceted by model type to compare results from various model configurations. NB indicates the negative binomial model, and ZINB indicates the zero-inflated negative binomial model

**Fig. 3:**
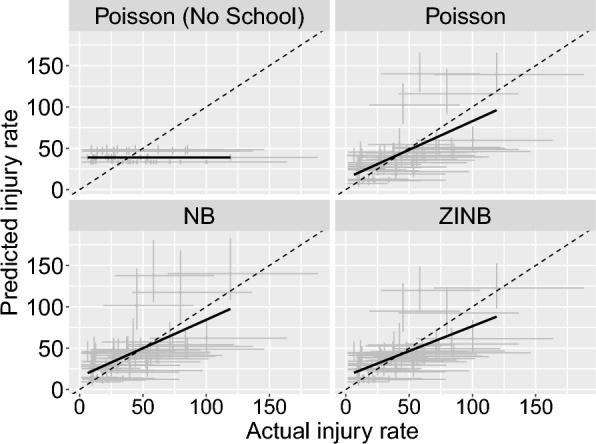
Actual and posterior predicted injury rates for sprain diagnoses reported during competition events in Division I men’s soccer. The y-axis shows the predicted injury rate and the x-axis shows the actual injury rate. The dashed line represents the line of equality, while the solid line is the linear regression fit (fitted by regressing the posterior medians on the actual injury rates). Error bars indicate the 90% credible intervals for both actual and predicted injury rates. The figure is faceted by model type to compare results across various model configurations. NB indicates the negative binomial model, and ZINB indicates the zero-inflated negative binomial model

**Fig. 4:**
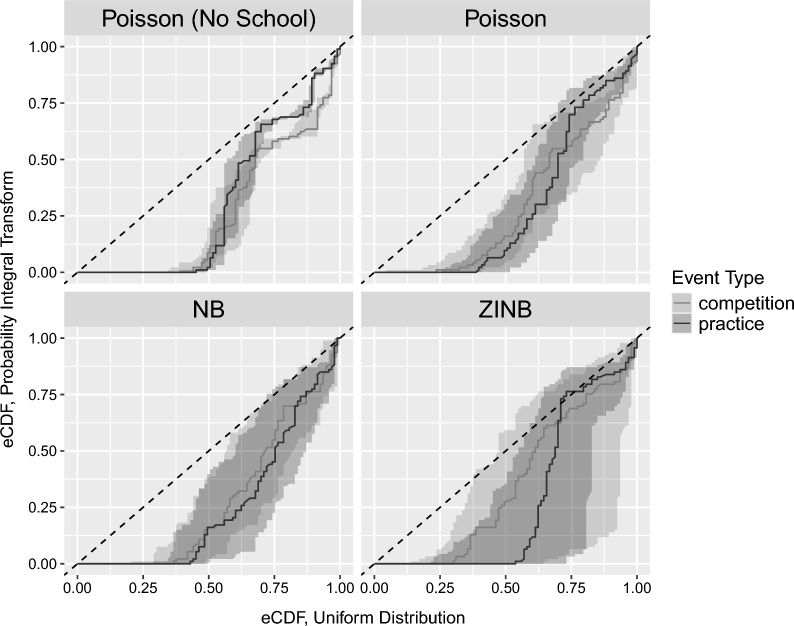
The plot illustrates the empirical cumulative distribution functions (eCDFs) of observed and simulated data for concussion diagnoses reported in Division I women's soccer. The x-axis represents the eCDF of a uniform distribution, and the y-axis represents the eCDF of the PIT-transformed data. Shaded ribbons indicate the 95% credible intervals for the simulated data, while solid lines represent the median PIT values. The dashed line represents the line of equality. The figure is faceted by model type to compare results across various model configurations. NB indicates the negative binomial model, and ZINB indicates the zero-inflated negative binomial model

We chose a prior for the school effects, $\zeta$, of the form: $\sim Normal(0, {\sigma }_{\zeta })$, and ${\sigma }_{\zeta }$ was set a prior of $\sim Cauchy(0, 10)$, which was constrained with a lower bound of 0 (effectively resulting in a half-$Cauchy$ distribution)– again, these allow for a large possible range of effect sizes. This modification yielded noticeable improvements in our model fit. This model generated zero injury cases in a manner comparable to the observed data (Fig. 2). Additionally, the Poisson model with the school factor included predicted injury rates that aligned more closely with the observed rates (Fig. 3). The probability integral transforms still showed a relatively poor fit for this model, and we subsequently considered a negative binomial model of the form:

6 $${X}_{i} \sim NB({\lambda }_{i}, \kappa )$$

where ${\lambda }_{i}$ indicates the mean count as determined through Eq. (5), and $\kappa>0$ is the overdispersion parameter, such that the variance is given by ${\lambda }_{i}+ \frac{{\lambda }_{i}^{2}}{\kappa }$, which exceeds that from a Poisson model. The negative binomial model outperformed the Poisson models across various posterior predictive checks. The negative binomial model handled extreme values better than the Poisson models (Fig. 1); it performed similarly to the Poisson”school” model in terms of the zero case count check and the actual vs. predicted injury rate check (Figs. 2, 3). However, the probability integral transform check, whilst improved as compared with the Poisson models, still was imperfect (Fig. 4).

Given the highly over-dispersed and zero-inflated nature of these data, we then considered a zero-inflated negative binomial model of the form:

7 $${X}_{i} \sim {ZINB}_{{\theta}_{j[i]}}({\lambda }_{i}, \kappa)$$

where ZINB represents a zero-inflated negative binomial model where ${\theta }_{j[i]}$ is the zero-inflated probability parameter $j\left[i\right]\in [competition, practices]$, which leads to an excess of zeros relative to the negative binomial model. The zero-inflated model performed comparably, perhaps slightly better than the NB model in posterior predictive checks. The zero-inflated formulation generated mean predicted rates that aligned closely with observed rates (Fig. 3) and performed similarly to the negative binomial model in accurately reflecting the proportion of zero injury cases (Fig. 2) and accounting for extreme values (Fig. 1). The Probability Integral Transforms (PIT) plots indicated that the zero-inflated model was comparably calibrated to the negative binomial model, while both models outperformed the Poisson models in mimicking the distributions of observed data (Fig. 4).

### Model comparison using leave-one-out-cross-validation

An alternative approach to choosing between models is to assess their predictive performance on out-of-sample data, and we also performed such tests on our four models. In alignment with our posterior predictive checks, the zero-inflated negative binomial model performed comparably to the negative binomial model and better than the Poisson models. This was corroborated by additional diagnostic measures– specifically Leave-One-Out Cross-Validation (LOO-CV) assessments. As shown in Supplemental Table 1, the Expected Log Pointwise Predictive Density (ELPD) values indicated that the ZINB and NB models offered similarly strong fits for the data in both men’s and women’s soccer cases, with both outperforming the Poisson models. ELPD serves as a representation of the out-of-sample predictive accuracy of a model, where higher values of ELPD (i.e., values approaching 0) are indicative of better fitting models. The minimal ELPD differences between the ZINB and NB models (Supplemental Table 1) highlight their comparable performance, with the ZINB model showing slight advantages in some areas.

**Table 1:** Sample summary of parameter estimates obtained from Negative Binomial model

Parameter	Men’s Soccer			Women’s Soccer		
	**Bayesian model**					
	**Mean**	**2.5%**	**97.5%**	**Mean**	**2.5%**	**97.5%**
**Competition (vs. Practice)**	1.28	1.16	1.40	1.33	1.23	1.44
**Division II (vs. Division I)**	−0.18	−0.57	0.22	0.01	−0.30	0.32
**Division III (vs. Division I)**	−0.03	−0.46	0.39	0.09	−0.26	0.43
**Diagnosis (Strain vs. Sprain)**	−0.02	−0.18	0.14	−0.07	−0.21	0.07
**Diagnosis (Contusion vs. Sprain)**	−0.23	−0.40	−0.07	−0.68	−0.83	−0.54
**Diagnosis (Concussion vs. Sprain)**	−1.41	−1.63	−1.20	−0.99	−1.14	−0.82

	**Frequentist model**					
	**Estimate**	**2.5%**	**97.5%**	**Estimate**	**2.5%**	**97.5%**
**Competition (vs. Practice)**	1.28	1.16	1.40	1.33	1.22	1.44
**Division II (vs. Division I)**	−0.17	−0.54	0.19	0.01	−0.29	0.31
**Division III (vs. Division I)**	−0.02	−0.42	0.37	0.09	−0.25	0.42
**Diagnosis (Strain vs. Sprain)**	−0.02	−0.18	0.13	−0.07	−0.20	0.07
**Diagnosis (Contusion vs. Sprain)**	−0.23	−0.39	−0.07	−0.68	−0.83	−0.53
**Diagnosis (Concussion vs. Sprain)**	−1.41	−1.62	−1.20	−0.99	−1.15	−0.83

Note: Estimates obtained from two distinct modeling approaches: the final Bayesian NB model and the analogous frequentist NB model. For the Bayesian model, the “2.5%” and “97.5%” columns represent the bounds of the 95% credible intervals, which summarize posterior uncertainty. In contrast, for the frequentist NB model, these columns represent the bounds of the 95% confidence intervals, summarizing the uncertainty in parameter estimates under repeated sampling assumptions

### Model results and interpretation

Following a comprehensive assessment of all model evaluations, considering relative model performance and ease of interpretation, we used a negative binomial model. While the ZINB model performed comparably to (and on occasion marginally better) than the NB model, we favored the NB model for its relative parsimony and more straightforward interpretation of model parameters (given the 2-stage interpretation involved in ZINB cases). A summary of each of the parameter posterior distributions from this model, can be found in Table 1. These results offer a contextual foundation for deriving interpretations that are akin to those obtained within the classical frequentist framework. For instance, the results associated with the $\alpha$ parameter indicate the relative rate of injuries in competition events compared with practice events, expressed on a log scale. The event type (competition) parameter from the women’s soccer model suggests that, on average, the log injury rate during competitions is 1.33 times the log rate during practice sessions, when controlling for covariate effects (a comparison of competition effect estimates across all model parameterizations is presented in Supplemental Fig. 3). Following this, we see that the average injury rate is 3.8 times as high in competitions as in practices based on our final model (i.e., $\text{exp}(1.33))$). A similar approach may be taken with all parameter estimates included in Table 1. Also included in Table 1 are parameter estimates derived from fitting the same model using a frequentist approach (i.e., we fit the identical negative binomial model with a random effect for school using the frequentist framework). While the parameter and interval estimates from both approaches are similar, this is attributable to our selection of weakly informative priors in the Bayesian framework—consequently, parameter estimates from the Bayesian analysis are predominantly data-driven (in Supplemental Fig. 4, we illustrate the similarity in actual vs. predicted results between the two approaches for the same scenario presented in Fig. 3; however, the comparison also demonstrates that the Bayesian negative binomial model provides better predictive performance). That said, there are notable distinctions in how the respective uncertainty intervals should be interpreted. Specifically, credible intervals from our Bayesian analysis indicate a 95% probability that the competition injury rate in women’s soccer is between 3.4 and 4.2 times as high as the practice rate. Such a direct probabilistic interpretation is not possible using the frequentist approach. Instead, the analogous 95% confidence interval suggests that if the same analyses were repeated 100 times, approximately 95 of these similarly constructed intervals would contain the true differential rate (between competitions and practices). Thus, the frequentist confidence interval is not a direct probability statement about the differential rate estimate but rather reflects long-term properties under repeated sampling.

### Limitations

It is important to acknowledge several limitations and challenges associated with the work presented here. Primary among these relate to the limitations inherent to sport injury surveillance data. The nature of injury surveillance data collection often results in a lack of detail in the captured data. As such, we acknowledge that various athlete, school-level, and contextual factors, which may contribute to injury rates, are not captured within the scope of this surveillance. Ascertainment of at-risk exposure time also presents a significant challenge in sports injury surveillance [23]. While the expression of exposure time in terms of AEs provides an efficient reporting solution, it is important to note that AEs are not the most precise measure of exposure time. As a consequence, the precision of injury incidence estimates may be compromised. Furthermore, while the methods of the NCAA ISP strive to standardize reporting practices, the system relies on the expertise of athletic trainers in reporting. This reliance may lead to variation in reporting practices between athletic trainers and potential non-differential misclassification of diagnoses. From an analytical standpoint, it is also important to recognize that our proposed approach, while systematic in its approach to model selection and specification, still makes assumptions regarding the underlying rate distributions within the negative binomial framework. Moreover, the selection of priors in Bayesian inference is inherently a subjective determination. While we opted for weakly informative priors for our parameters of interest in this study, different investigators may choose different prior specifications and ultimately observe different results. It should also be noted that the application of Bayesian frameworks can be computationally intensive, especially with large datasets and complex models such as those used in the present study.

## Summary and considerations

**Fig. 5:**
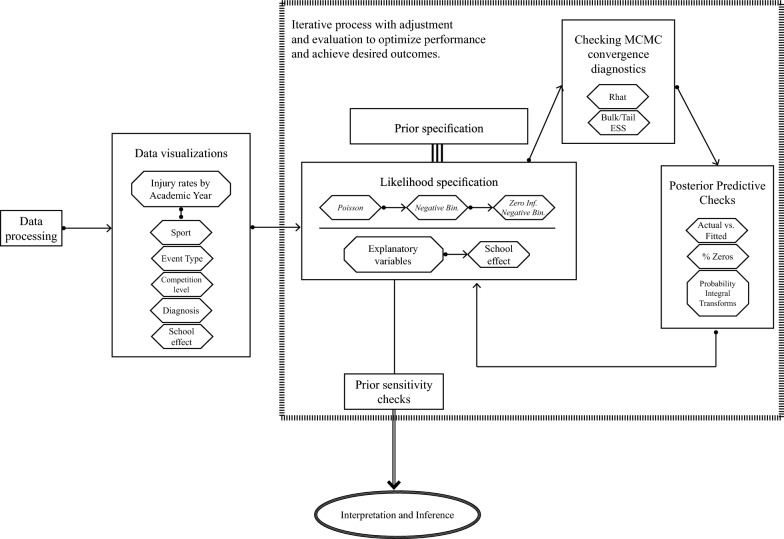
Sample workflow describing analytical approach for Bayesian estimations of injury rate using sport injury surveillance data [18]

The Bayesian approach offers a conceptual parallel to traditional methods for estimating injury rates in sports injury epidemiology. We demonstrate the application of the Bayesian approach to estimate injury rates across various strata of covariates using data obtained from a comprehensive and longitudinal sport injury surveillance system spanning a period of five years. In Bayesian inference, like in more traditional approaches, it is important to follow best practices to ensure that given analyses are robust and reliable. In Bayesian inference, the requirement to set and justify choices of prior distributions, and the demands of assessing model fit are if anything higher than in traditional analyses owing to the ways that the models are fitted computationally and how these can go wrong (e.g. lack of convergence of MCMC). In Fig. 5 we provide the sketch of a framework for performing a Bayesian analysis. Additionally, supplementary code resources are provided to aid investigators in adapting and implementing the Bayesian methodology to their own datasets. Throughout the iterative process outlined, we highlight key decision points and discuss the rationale behind our choices and evaluations. Like in most real-life analyses, our final chosen model did not perfectly fit our data according to all model checks. Indeed, we selected this more parsimonious model over a more complex alternative that offered only marginal improvements in fit. We also believed that the NB model would be more readily adaptable to other injury surveillance contexts, where data are often aggregated and do not support the individual-level structure required by zero-inflated models. Nonetheless, this analysis highlights the challenges of modeling real data, and the inevitable trade-offs required in this process. The modeling framework we describe supports the analyst to make an informed decision over model choice.

We now discuss what we believe are key benefits of using a Bayesian framework. The Bayesian approach considers injury counts as outcomes resultant of an underlying probabilistic process, in contrast to traditional methods which estimate injury rates as simple ratios of observed injuries to measured exposures. By explicitly modeling the underlying probabilistic process, the Bayesian approach inherently captures uncertainty in injury rate estimates, a feature typically not directly addressed by traditional methods. The Bayesian approach also allows for the estimation of plausible injury rates even when data are sparse, because prior information is used to inform estimates. Moreover, Bayesian analysis involves a simulation-based approach for obtaining the estimates; this approach offers additional advantages from both analytical and inferential perspectives– notably providing considerable flexibility in evaluating model fidelity and fit. As demonstrated above, we did not make a priori decisions on how to assess our models, and rather relied on creative graphical approaches (ex. checking the proportion of zeros or using PITs) to evaluate our models. Finally, it may also be acknowledged that successful implementation of Bayesian inference required the development of customized software packages allowing for the application of MCMC for analysis. This affords substantial flexibility in approach and modeling, and the ability to model complex processes in creative manners. The package used in the present analysis– Stan, also boasts a very active user forum, which also may be leveraged for troubleshooting problems that may arise during analysis.

Collectively, we reemphasize that the methodology described in this study provides an alternative approach for researchers involved in estimating injury rates based on similar data. While we have highlighted several advantages of this approach, we acknowledge that traditional estimation methods also possess their own merits in various aspects. Our workflow and accompanying code are presented here to assist researchers in implementing this methodology in their specific research scenarios [18]. There is also a need for further investigation and application of these methods using diverse samples and different types of data in order to comprehensively assess their value in the field of injury epidemiology.

## Supplementary Information

Additional file1

## Data Availability

Due to the nature of the research, supporting data are not available for deposit.

## Code availability

Code used for analysis are contained within the supplemental document.
