# Supplementary material for: Bayesian methods for estimating injury rates in sport injury epidemiology
Source: Inj Epidemiol. 2025 Jun 6;12:31. doi: 10.1186/s40621-025-00583-z (PMC12142926; doi:10.1186/s40621-025-00583-z)
Supplement: Supplementary file 1 — Additional file1 [file 40621_2025_583_MOESM1_ESM.pdf]

Bayesian Methods for Estimating Injury Rates in Sport Injury Epidemiology: Suppelemental Materials

2025-04-01

Supplemental Figures

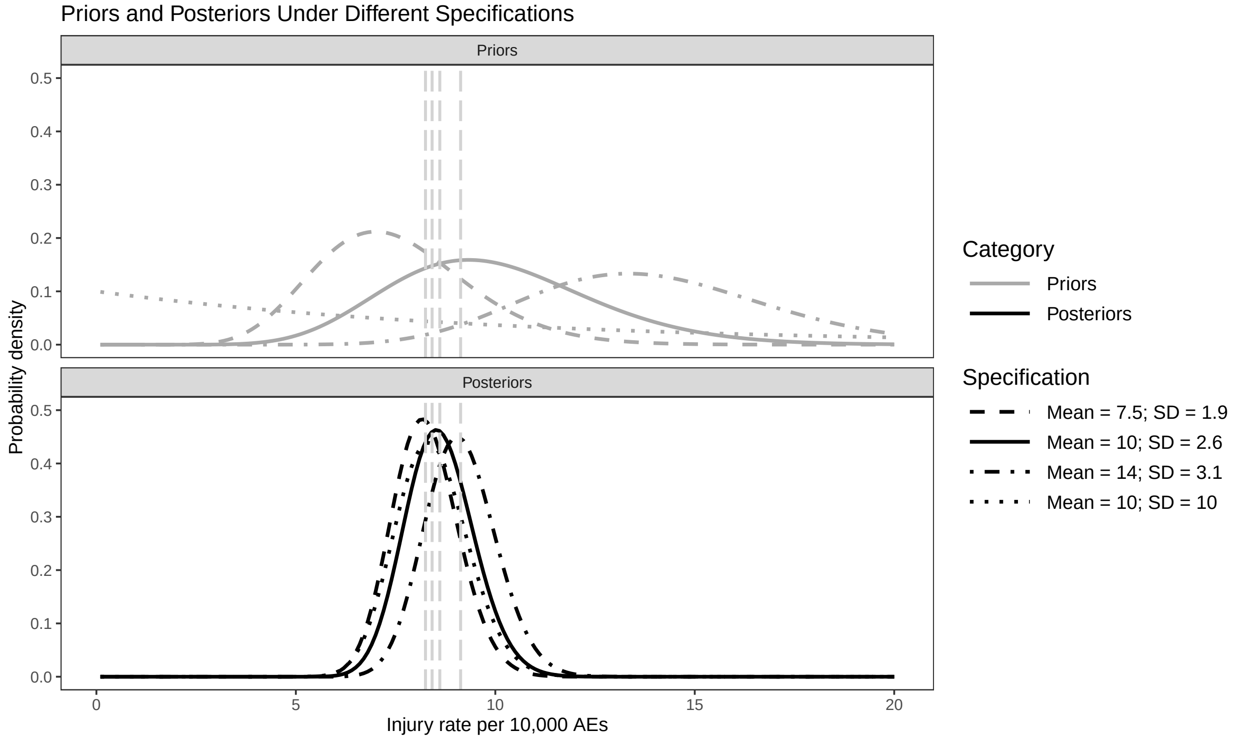

Supplemental Figure 1. The effect of priors on posteriors for an injury rate. The top panel displays four prior distributions over the injury rate, each with different assumptions about the expected rate and uncertainty. These include priors with means of 7.5, 10.0, and 14.0 and corresponding standard deviations of 1.9, 2.6, and 3.1, respectively, as well as a weakly informative prior with a much flatter shape (mean = 10.0; SD = 10.0). The bottom panel shows the resulting posterior distributions when each prior is combined with the same observed data. Vertical dashed gray lines in the posterior panel indicate the posterior mean for each specification. Despite the differences in the shape and informativeness of the priors, the posterior distributions are similar—illustrating that, in this example, the observed data are sufficiently informative to dominate the influence of the prior.

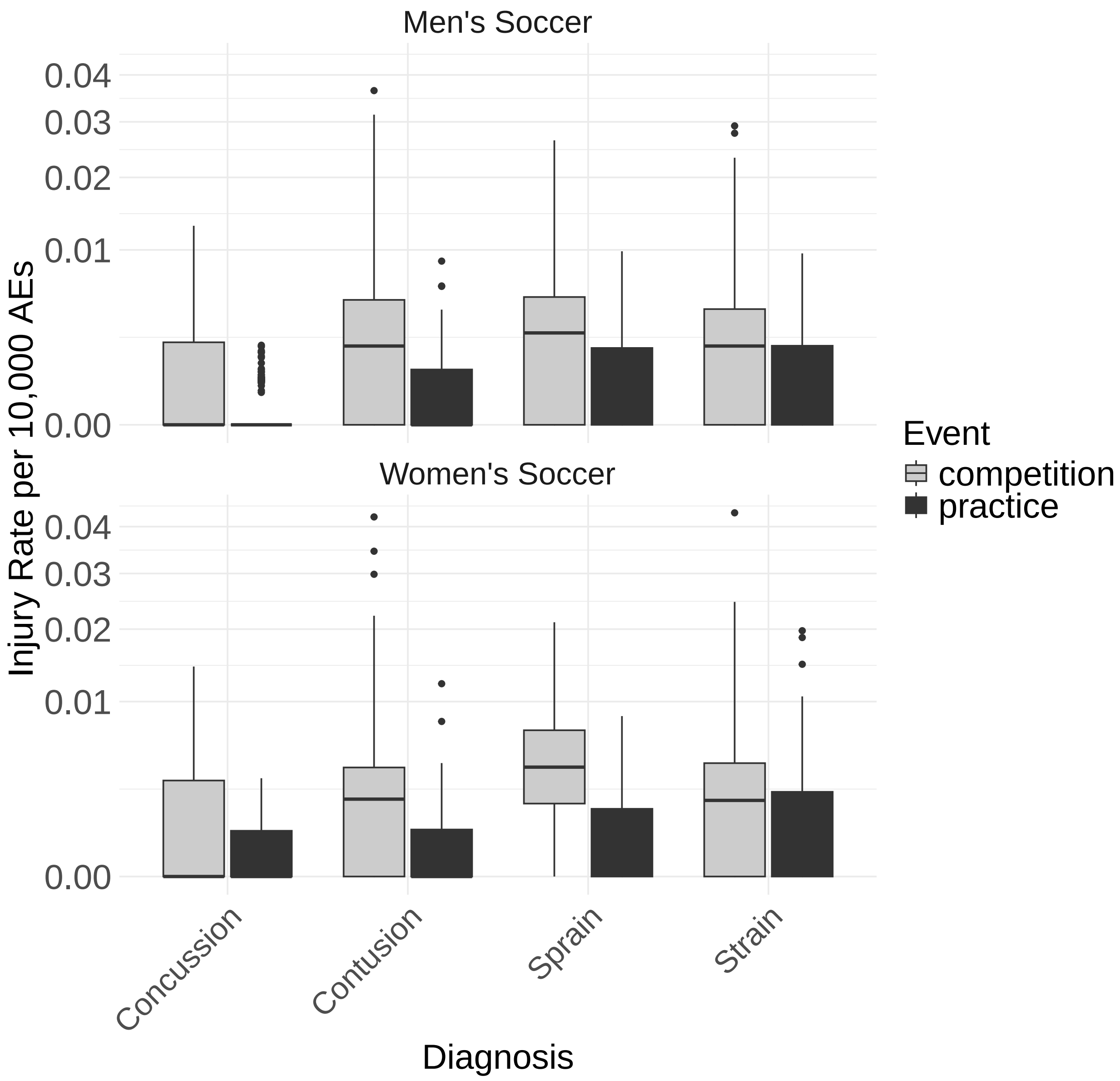

Supplemental Figure 2. Raw injury rates for men's and women's soccer across diagnoses, stratified by event types. Each boxplot represents the spread of injury rates for a specific diagnosis, with the vertical axis indicating the injury rate and the horizontal axis representing the diagnosis categories. The vertical axis is scaled using a square root transformation. The boxes display the interquartile range (IQR), with the median marked by a horizontal line inside each box.

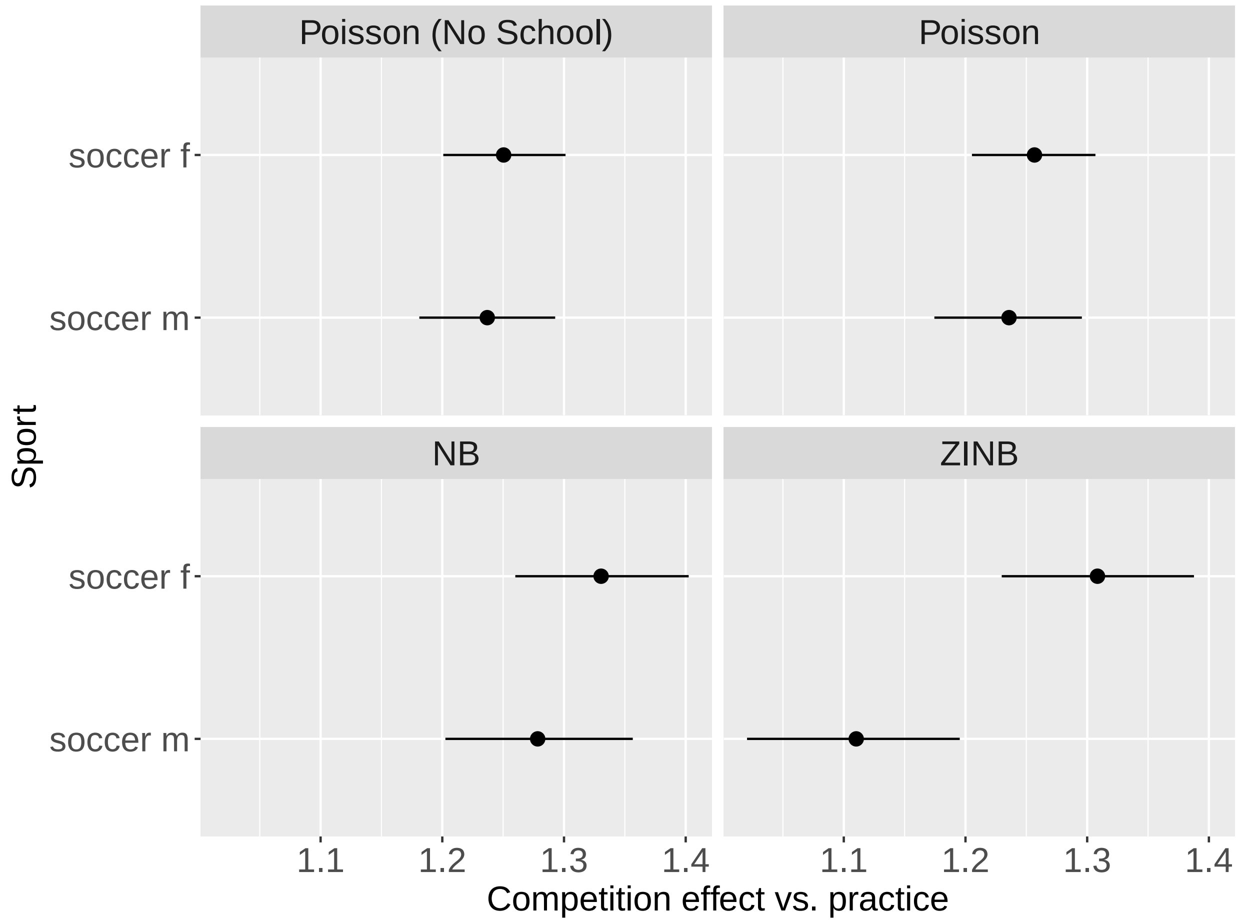

Supplemental Figure 3. Plot illustrates the posterior estimates of competition effects versus practice for men's and women's soccer from all models considered. Each point represents either men's soccer or women's soccer, with the x-axis showing the median estimate and the error bars indicating the 10th and 90th percentiles. The figure is faceted by model type to compare results across different model configurations. NB indicates the negative binomial model, and ZINB indicates the zero-inflated negative binomial model.

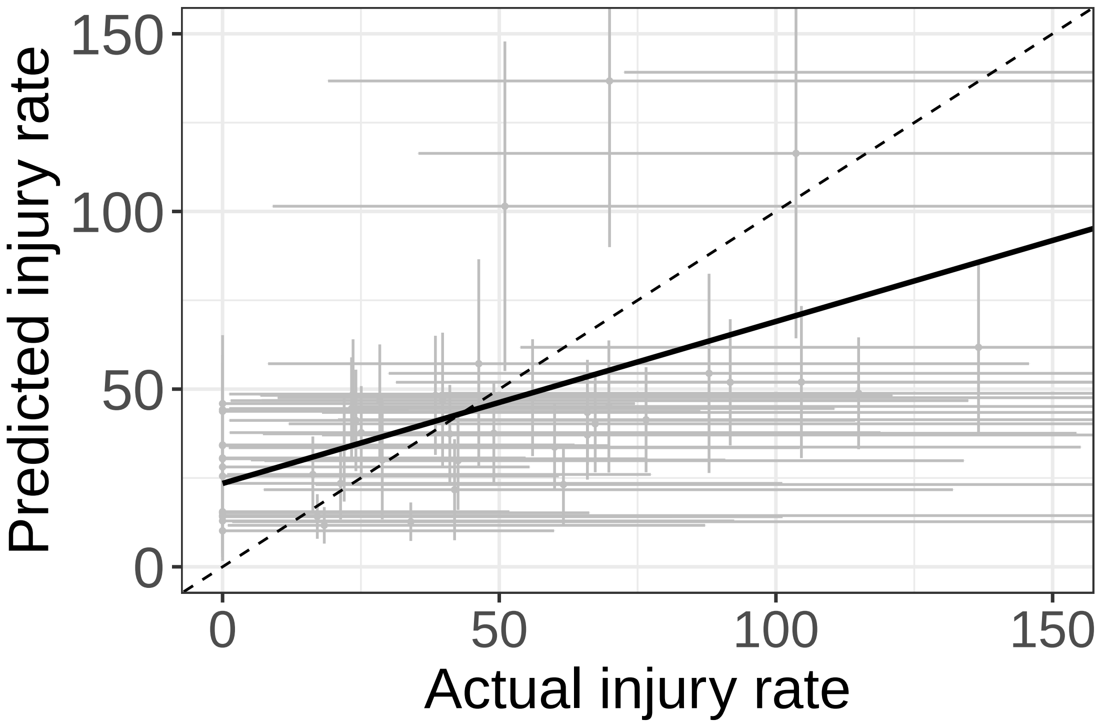

Supplemental Figure 4. Actual and predicted injury rates for sprain diagnoses reported during competition events in Division I men's soccer, based on the frequentist negative binomial model. The y-axis shows the predicted injury rate and the x-axis shows the actual injury rate. The dashed line represents the line of equality, while the solid line is the linear regression fit (fitted by regressing the predicted rates on the actual injury rates). Error bars represent 90% confidence intervals for both actual and predicted injury rates.

Supplemental Tables

Supplemental Table 1. Leave-one-out-cross-validation for Men's and Women's Soccer and Summary of Parameters

Metric	Men's Soccer				Women's Soccer			
	ZINB	NB	Poisson	Poisson (No school)	ZINB	NB	Poisson	Poisson (No school)
**ELPD difference**	0.00	-9.74	-76.24	-505.69	0.00	-9.76	-143.53	-672.19
**SE difference**	0.00	7.41	16.45	51.55	0.00	8.81	23.59	88.30

Compares different statistical models considered in data analysis. NB indicates the negative binomial model and, ZINB indicates the zero-inflated negative binomial model. The ELPD difference column shows the difference in expected log predictive density between each model and the best-performing model. A value of 0 indicates the best model, while negative values indicate how much worse other models perform. The SE difference column represents the standard error of the ELPD difference, quantifying the uncertainty in the ELPD difference estimate.

Supplemental Code

- The Bayesian framework allows for flexible estimation of injury rates by incorporating prior information and handling overdispersion in injury counts. This approach yields realistic results even in cases of sparse or variable data. Bayesian inference involves drawing samples from the posterior distribution to represent the range of plausible values for each parameter.
- The negative binomial model was determined to be an appropriate choice for estimating injury rates from sports injury surveillance data in our analysis. The negative binomial model in our analysis was implemented using the Stan program noted below.

```
data{
  int N; //number of observations
  int event[N]; //unique event identity
  int inj[N]; //unique injuries for each observation
  int div[N]; //unique division identity for each observation
  real aes_scaled[N]; //unique AEs for each observation
  int diag[N]; //unique diagnosis for each observation
  int ndiv; //total number of divisions
  int ndiag; //total number of diagnoses
  int year[N]; //unique year identity for each observation
  int nyear; //total number of years
  int eventyear[N]; //unique event, year identifiers
  int neventyear; //total number of event, year combinations
  int divdiag[N]; //unique division, diagnosis identity
  int ndivdiag; //total number of div, diag combinations
  int yeardiag[N]; //unique year, diagnosis identifiers
  int nyeardiag; //total number of year, diagnosis combinations
  int divevent[N]; //unqiue division, event identifiers
  int ndivevent; //total number of division, event combinations
  int nschools; //total number of schools supplying data
  int school[N]; //unique school identity for each observation
  int N_sim; //number of contrasts
  int event_sim[N_sim]; //unique event identity for each observation
  int div_sim[N_sim]; //unique div identity for each observation
  int diag_sim[N_sim]; //unique diagnosis identity for each observation
  int year_sim[N_sim]; //unique event identity for each observation
  int divdiag_sim[N_sim]; //unique division, diagnosis identity for each observation
  int yeardiag_sim[N_sim]; //unique year, diagnosis identity for each observation
  int divevent_sim[N_sim]; //unique division, event identity for each observation

}


parameters{
  real gamma; //intercept in model
  real alpha; //beta for event
  real beta[ndiv]; //beta for division (unique for each division)
  real epsilon[ndiag]; //beta for diagnosis
  real kappa[nyear]; //beta for year
  real zeta[nschools]; //specifies estimate unique to each school
  real<lower=0> sigma_zeta; //specifies amount of school-level variation
  real<lower=0> phi; //overdispersion parameter
}
```

```
transformed parameters{
  real inj_rate[N]; //Defines inj_rate as a 1 dimensional array of length N
  for (i in 1:N){ //For each observation loop and estimate injury rate
    inj_rate[i]= exp(gamma + alpha*event[i] + beta[div[i]] + epsilon[diag[i]] +
    kappa[year[i]] + zeta[school[i]]); //Injury rate defined using this equation

  }
}

model{

  for(i in 1:N)
  inj[i] ~ neg_binomial_2(inj_rate[i]*aes_scaled[i], phi);

  //Setting priors for each parameter
  gamma ~ cauchy(0,10);
  alpha ~ cauchy(0,10);
  beta ~ cauchy(0,10);
  epsilon ~ cauchy(0,10);
  kappa ~ cauchy(0,10);
  zeta ~ normal(0, sigma_zeta);
  sigma_zeta ~ cauchy(0,10);
  phi ~ cauchy(0, 10);
}


generated quantities {
  vector[N] inj_realised;
  vector[N] y_unif;
  vector[N] log_likelihood;
  real inj_rate_sim[N_sim];
  for (i in 1:N_sim){
    inj_rate_sim[i]= exp(gamma + alpha*event_sim[i] + beta[div_sim[i]] +
    epsilon[diag_sim[i]] + kappa[year_sim[i]] + zeta[school[i]]);
  }

  for (i in 1:N){
    y_unif[i] = neg_binomial_2_cdf(inj[i], inj_rate[i] * aes_scaled[i], phi);
    inj_realised[i] = neg_binomial_2_rng(inj_rate[i] * aes_scaled[i], phi);
    log_likelihood[i] = neg_binomial_2_lpmf(inj[i] | inj_rate[i] * aes_scaled[i], phi);
  }
}
```

- The Stan model was fit using the RStan package, with data passed as a list containing total injuries and total exposures. No U-Turn Sampling was conducted with 2000 iterations across four chains. Posterior distributions were extracted from the model fit to further analyze injury rates, and perform predictive checks for model validation.
